# Conserved miR-26b enhances ovarian granulosa cell apoptosis through HAS2-HA-CD44-Caspase-3 pathway by targeting HAS2

**DOI:** 10.1038/srep21197

**Published:** 2016-02-18

**Authors:** Jiying Liu, Fei Tu, Wang Yao, Xinyu Li, Zhuang Xie, Honglin Liu, Qifa Li, Zengxiang Pan

**Affiliations:** 1 College of Animal Science and Technology, Nanjing Agricultural University, Nanjing 210095, People’s Republic of China

## Abstract

The hyaluronan synthase 2 (HAS2)-hyaluronic acid (HA)-CD44-Caspase-3 pathway is involved in ovarian granulosa cell (GC) functions in mammals. HAS2 is a key enzyme required for HA synthesis and is the key factor in this pathway. However, the regulation of HAS2 and the HAS2-mediated pathway by microRNAs in GCs is poorly understood. Here, we report that miR-26b regulates porcine GC (pGC) apoptosis through the HAS2-HA-CD44-Caspase-3 pathway by binding directly to the 3′- untranslated region of HAS2 mRNA. Knockdown of miR-26b reduced pGC apoptosis. Luciferase reporter assays demonstrated that HAS2 is a direct target of miR-26b in pGCs. Knockdown and overexpression of miR-26b increased and decreased, respectively, HA content, and HAS2 and CD44 expression in pGCs. At the same time, inhibition and overexpression of miR-26b decreased and increased the expression of Caspase-3, a downstream factor in the HAS2-HA-CD44 pathway. Moreover, knockdown of HAS2 enhanced pGC apoptosis, reduced the inhibitory effects of a miR-26b inhibitor on pGC apoptosis, repressed HA content and CD44 expression, and promoted Caspase-3 expression. In addition, overexpression of HAS2 has a opposite effect. Collectively, miR-26b positively regulates pGC apoptosis via a novel HAS2-HA-CD44-Caspase-3 pathway by targeting the HAS2 gene.

Hyaluronic acid (HA), an extracellular glycosaminoglycan, is expressed in almost all mammalian systems. In mammalian ovaries, HA is expressed in cumulus cells, is an important factor for cumulus expansion and oocyte maturation^1^, and prevents women’s diseases such as primary ovarian insufficiency^2^. In the ovary, HA inhibits human follicular granulosa cell (GC) apoptosis^3^, consistent with its function in porcine GC (pGC) apoptosis^4^. Inhibition of ovarian HA synthesis with chemicals, such as deoxynivalenol, induces follicular atresia, and addition of Streptomyces hyaluronidase, which specifically degrades HA, induces the arrest of follicle growth in an *in vitro* culture system^5^. Likewise, the anti-apoptotic role of HA is evident in other cells, such as skin fibroblasts^6^ and smooth muscle cells^7^. HA is anchored to the cell membrane surface by the HA-binding receptor CD44, and activation or inhibition of HA affects the mRNA level of CD44 in pGCs^4^. Meanwhile, HA and CD44 both regulate the expression of apoptosis marker genes, including Caspase-3, -8, and -9, while an anti-CD44 antibody prevents the anti-apoptotic effect of HA in GCs^3^,^4^, indicating that HA suppresses GC apoptosis via the CD44-Caspase axis.

HA is synthesized directly on the plasma membrane by enzymes termed hyaluronan synthases (HASs)^8^. Three HAS isoforms (HAS1, HAS2, and HAS3) have been identified in mammals, of which HAS2 is the most active and highly expressed^9^. HAS2 is reportedly the most common and widely functioning HAS in mammals^8^. Knockout studies in mice showed that Has2^−/−^ embryos lack HA, resulting in embryonic lethality during mid-gestation^10^,^11^. HAS2 has a close relationship with ovary development and function, and is predominantly located in the GC layers of rat and sheep ovaries^5^,^12^. Knockdown of CCAAT-enhancer-binding protein CEBβ decreases the HAS2 mRNA level and results in the arrest of pGCs at S phase of the cell cycle, indicating that HAS2 plays an important role in GC function^13^. Recent studies found that the HAS2 mRNA level is lower in GCs of polycystic ovary syndrome (PCOS) patients than in those of controls^14^, and is highly upregulated in human GCs treated with human chorionic gonadotropin (hCG)^15^. Moreover, HAS2 is upregulated during expansion of the cumulus-oocyte complex, and knockdown of HAS2 decreases the degree of cumulus expansion; therefore, HAS2 is regarded as a marker of cumulus expansion and ovulation^16^,^17^,^18^. However, there is no direct proof that HAS2 participates in GC apoptosis.

In mammals, HAS2 is regulated by multiple factors such as hormones, transcription factors, and epigenetic factors^17^,^18^,^19^. In GCs, follicle-stimulating hormone (FSH) stimulates HAS2 expression^12^ and affects downstream events of the HAS2-HA-CD44-Caspase-3 pathway such as HA synthesis, CD44 expression, and Caspase-3 expression and activity^4^,^17^. Transcription factors such as CREB1, RAR, SP1, SP3, and YY1 regulate HAS2 expression by binding to functional binding sites within the HAS2 promoter region^20^. Epigenetic modifications such as DNA methylation^17^, histone acetylation^21^, O-GlcNAcylation^22^, and microRNA (miRNA) mechanisms^23^ are also involved in the regulation of HAS2 expression, among which miRNA mechanisms are the recently most studied. miRNAs function in mRNA degradation and gene translation repression and play an important role in complex biological activities^24^. Recently, increasing numbers of studies have focused on the regulation of HAS2 by miRNAs^25^,^26^. However, there are no studies regarding miRNA regulation of HAS2 and the HAS2-HA-CD44-Caspase-3 pathway in mammalian ovaries. Here, we focused on the epigenetic regulation of HAS2 and the HAS2-HA-CD44-Caspase-3 pathway by miRNAs in pGCs. The results show that miR-26b regulates pGC apoptosis and follicular atresia through the HAS2-HA-CD44-Caspase-3 pathway by directly targeting HAS2. Our findings aid understanding of the epigenetic regulation of HAS2 and elucidation of the miR-26b regulation network.

## Results

### Identification and characterization of the pig miR-26b gene

**Figure 1:**
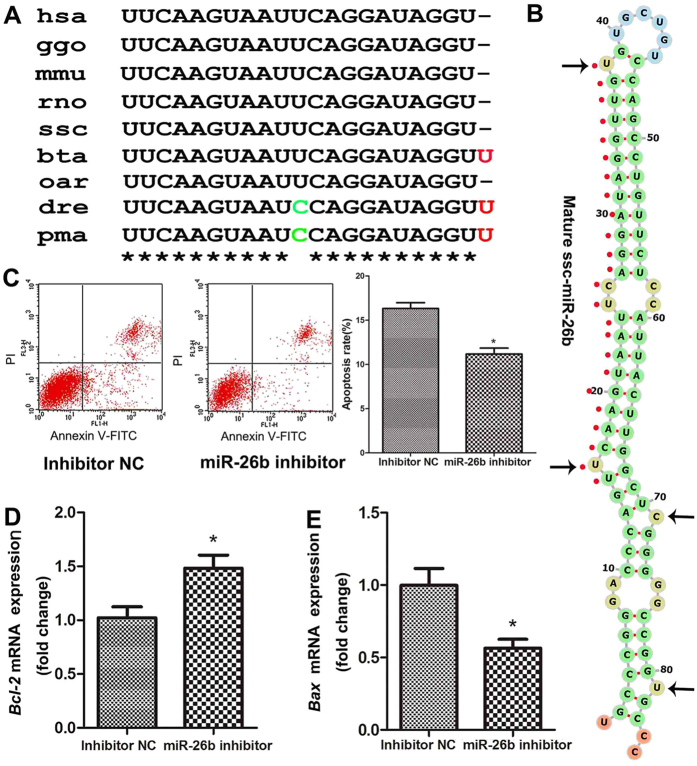
Inhibition of miR-26b reduces pGC apoptosis. (**A**) miR-26b mature sequences are highly conserved among vertebrates. Nucleotides that diverge from the human sequence are highlighted in red and green. hsa, human; ggo, Gorilla gorilla; mmu, Mus musculus; rno, Rattus norvegicus; ssc,Sus scrofa; bta, Bos Taurus; oar, Ovis aries; dre, Danio rerio; pma, Petromyzon marinus. (**B**) Stem-loop structures of pig pre-miR-26b predicted by the Vienna RNAfold webserver (http://rna.tbi.univie.ac.at/cgi-bin/RNAfold.cgi). Mature miRNA sequences are indicated by red spots. Black arrows indicate asymmetric bulges. (**C**) Knockdown of miR-26b inhibited pGC apoptosis. The pGC apoptosis rate after miR-26b inhibitor treatment was determined by Annexin V FITC-PI staining and FACS analysis. (**D**) Knockdown of miR-26b increased the mRNA level of the anti-apoptotic gene Bcl-2. (**E**) Knockdown of miR-26b decreased the mRNA level of the pro-apoptotic gene Bax. Gene expression levels were normalized to that of GAPDH. *p < 0.05.

We previously demonstrated that miR-26b is an important epigenetic regulator of pGC function and follicular atresia^27^. However, the gene encoding pig miR-26b is unknown. Here, we identified and characterized the gene encoding pig miR-26b, ssc-miR-26b. The precursor of the pig miR-26b gene is 85 bp in length. The nucleotide sequence is consistent with that in cattle and sheep and is highly homologous with that in other mammals (89.41% similar to human and 96.51% similar to mouse and rat) (Supplementary Fig. S1). The mature sequence of pig miR-26b is UUCAAGUAAUUCAGGAUAGGU, which is completely consistent with that in other mammals, while mature miR-26b in Actinopterygii contains a U → C mutation at nucleotide 11 (Fig. 1A). Meanwhile, the seed region (UCAAGUA) of miR-26b is conserved among vertebrate species. In addition, we identified four asymmetric bulges in the structure of ssc-miR-26b duplexes (Fig. 1B), which may affect the initiation of transitivity. These results indicate that the precursor and mature sequences of miR-26b are highly conserved and have similar functions in different species.

### Knockdown of miR-26b represses pGC apoptosis

To further confirm the role of miR-26b in pGC apoptosis, we knocked down miR-26b in pGCs cultured *in vitro* by transfecting a miR-26b inhibitor. miR-26b inhibitor treatment significantly reduced the rate of pGC apoptosis (Fig. 1C). Expression of the anti-apoptotic gene Bcl-2 and the pro-apoptotic gene Bax was detected in pGCs, and treatment with a miR-26b inhibitor significantly increased expression of the former (Fig. 1D) and significantly decreased expression of the latter (Fig. 1E). These data further confirm that miR-26b promotes pGC apoptosis and that knockdown of miR-26b represses pGC apoptosis.

### The HAS2 gene is a candidate target of miR-26b

**Figure 2:**
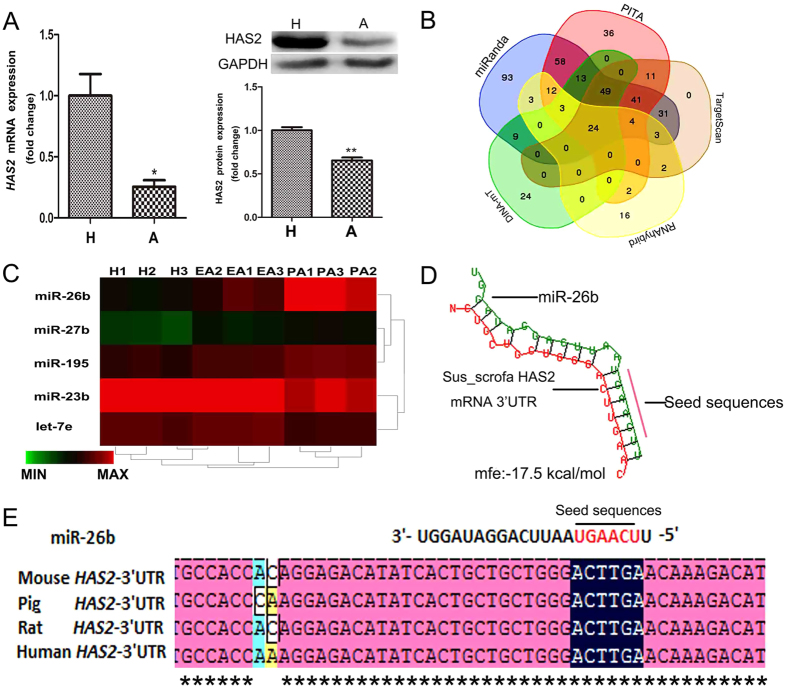
HAS2 is a candidate target gene of miR-26b. (**A**) HAS2 expression level in healthy follicles and atresia follicles. H: healthy follicles; A: atresia follicles. mRNA and protein levels were determined by RT-qPCR and western blot analyses, respectively. Cropped gels were used for our western blot results. (**B**) miRNAs that target the HAS2 gene were predicted by five algorithms: TargetScan, miRanda, PITA, DINA-mT, and RNAhybrid. Twenty-four miRNAs were commonly predicted. (**C**) Five miRNAs predicted to regulate HAS2 were differentially expressed during follicular atresia in porcine ovaries. H: healthy follicles; EA, early atresia follicular. PA, progressively atretic follicles. (**D**) The miR-26b-binding site within the 3′-UTR of HAS2 was predicted by RNAhybrid. (**E**) Alignments of miR-26b mature sequences and the 3′-UTR of HAS2 from pig and other mammals. Red letters indicate the seed sequences of miR-26b. Asterisks indicate complementarity. *p < 0.05, **p < 0.01.

miRNAs usually function by targeting multiple genes, even in the same biological process. In our analysis, HAS2, a key factor for follicular development and GC function in mammals ovaries^13^, was a predicted target gene of miR-26b, in addition to the ATM and SMAD4 genes, as previously reported^27^,^28^. Real-time quantitative PCR (RT-qPCR) and western blot analyses showed that the mRNA and protein levels of HAS2 were significantly downregulated during porcine ovarian follicular atresia (Fig. 2A), opposite to the changes in miR-26b expression. Meanwhile, 24 miRNAs that target HAS2 were commonly predicted by five algorithms: TargetScan, miRanda, PITA, DINA-mT, and RNAhybrid (Fig. 2B). Five of these were differentially expressed during porcine follicular atresia (Fig. 2C), and miR-26b was the most significantly elevated miRNA. To further verify that miR-26b regulates porcine HAS2, we identified a putative miR-26b-binding site at residues 125–130 of the 3′-untranslated region (UTR) of pig HAS2 using RNAhybrid (Fig. 2D). Comparison of the miR-26b seed sequence with the 3′-UTR of pig HAS2 and HAS2 in other vertebrates identified complementary pairing (Fig. 2E). These results suggest that the HAS2 gene is a candidate target of miR-26b.

### HAS2 is a direct target gene of miR-26b in pGCs

**Figure 3:**
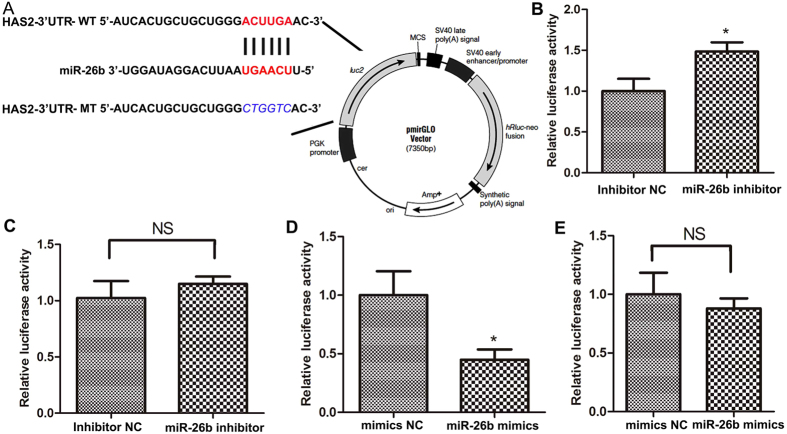
miR-26b directly regulates HAS2. (**A**) Putative miR-26b-binding sites (red letters) and mutated sites (blue italic letters) in the 3′-UTR of pig HAS2 in the pmirGLO Dual-Luciferase miRNA Target Expression Vector. (**B**) Luciferase activity assays of HeLa 229 cells co-transfected with HAS2 3′-UTR-WT and a miR-26b inhibitor. (**C**) Luciferase activity assays of HeLa 229 cells co-transfected with HAS2 3′-UTR-MT and a miR-26b inhibitor. (**D**) Luciferase activity assays of HeLa 229 cells co-transfected with HAS2 3′-UTR-WT and miR-26b mimics. (**E**) Luciferase activity assays of HeLa 229 cells co-transfected with HAS2 3′-UTR-MT and miR-26b mimics. The activity of the firefly luciferase protein was normalized to Renilla luciferase activity. *p < 0.05.

To determine whether HAS2 is a direct target of miR-26b, luciferase reporter plasmids harbouring the 3′-UTR of wild-type HAS2 (HAS2 3′-UTR-WT) and a seed region HAS2 mutant (HAS2 3′-UTR-MT) were constructed (Fig. 3A). HAS2 3′-UTR-WT and HAS2 3′-UTR-MT were co-transfected with a miR-26b inhibitor into HeLa 229 cells. Luciferase assays revealed that inhibition of miR-26b significantly increased the luciferase activity of HAS2 3′-UTR-WT (Fig. 3B), but did not change the luciferase activity of HAS2 3′-UTR-MT (Fig. 3C). In addition, overexpression of miR-26b by co-transfection significantly decreased the luciferase activity of HAS2 3′-UTR-WT (Fig. 3D), but did not change the luciferase activity of HAS2 3′-UTR-MT (Fig. 3E). All these results show that HAS2 is a direct target of miR-26b.

### miR-26b enhances pGC apoptosis via translational regulation of HAS2

**Figure 4:**
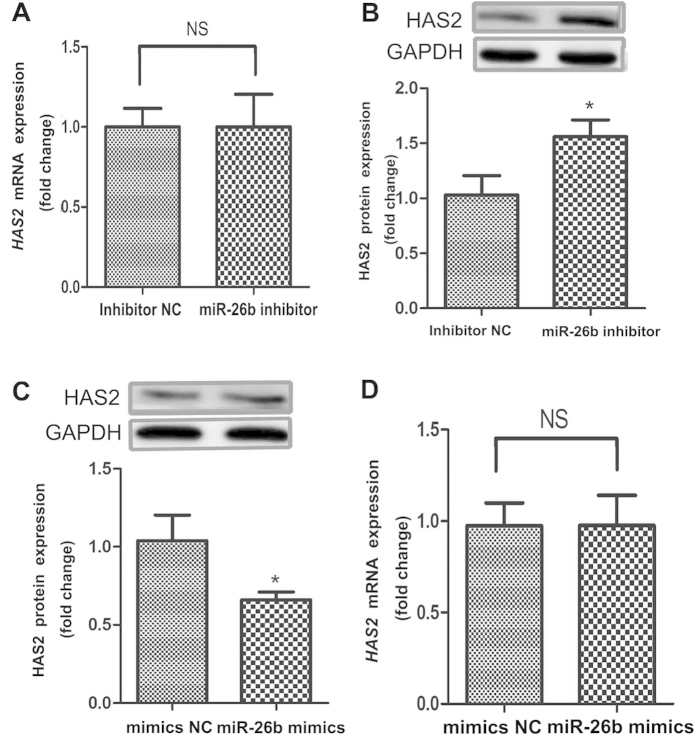
miR-26b inhibits HAS2 expression in pGCs. (**A**) The effect of miR-26b knockdown on the HAS2 mRNA level in pGCs. (**B**) Knockdown of miR-26b increased the HAS2 protein level in pGCs. (**C**) Overexpression of miR-26b decreased the HAS2 protein level in pGCs. (**D**) The effect of miR-26b overexpression on the HAS2 mRNA level in pGCs. *p < 0.05.

To further investigate the molecular mechanism by which miR-26b affects pGC apoptosis, we measured the mRNA and protein levels of HAS2 after miR-26b inhibitor treatment. The protein level, but not the mRNA level, of HAS2 was significantly upregulated in pGCs transfected with a miR-26b inhibitor (Fig. 4A,B). In addition, overexpression of miR-26b by co-transfection with miR-26b mimics significantly decreased the HAS2 protein level (Fig. 4C), but did not affect the HAS2 mRNA level (Fig. 4D). These data indicate that miR-26b promotes pGC apoptosis by targeting the HAS2 gene and inhibiting HAS2 translation.

### HAS2 inhibits pGC apoptosis

**Figure 5:**
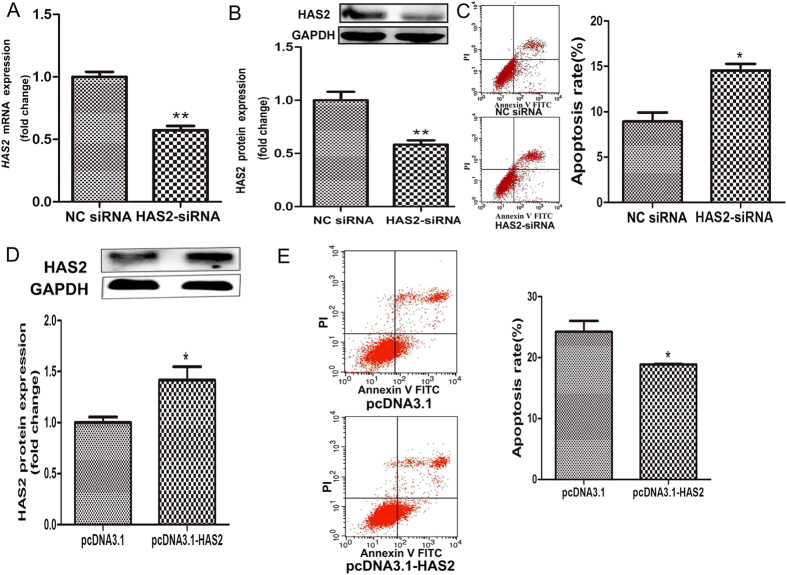
HAS2 inhibits pGC apoptosis. (**A**) mRNA expression in pGCs cultured *in vitro* and treated with HAS2-siRNA. (**B**) HAS2-siRNA treatment significantly decreased the HAS2 protein level in pGCs. (**C**) The pGC apoptosis rate was determined after HAS2-siRNA treatment. (**D**) pcDNA3.1-HAS2 treatment significantly increased the HAS2 protein level in pGCs. (**E**) The pGC apoptosis rate was determined after pcDNA3.1-HAS2 treatment. *P < 0.05, **P < 0.01.

HAS2 is involved in GC apoptosis and follicular atresia^14^,^29^. To further explore the effect of HAS2 on pGC apoptosis, we knocked down HAS2 in pGCs using RNA interference. RT-qPCR and western blot analyses revealed that treatment with HAS2-small interfering RNA (siRNA) significantly reduced the mRNA (Fig. 5A) and protein (Fig. 5B) levels of HAS2. However, two other HAS isoforms, HAS1 and HAS3, did not show compensatory increases in HAS2- silenced pGCs (Supplementary Fig. S2). The apoptosis rate was significantly higher in pGCs treated with HAS2-siRNA than in pGCs treated with negative control (NC) siRNA (Fig. 5C), suggesting that knockdown of HAS2 promotes pGC apoptosis. To further test the role of HAS2 in pGC apoptosis, we generated the porcine HAS2 overexpression vector, pcDNA3.1-HAS2, which increased the expression of HAS2 when transfected into pGCs, as shown by western blot analysis (Fig. 5D). The apoptosis rate was down-regulated by HAS2 overexpression (Fig. 5E). These results indicate that HAS2 inhibits pGC apoptosis.

### HAS2 regulates the HA-CD44-Caspase-3 pathway

**Figure 6:**
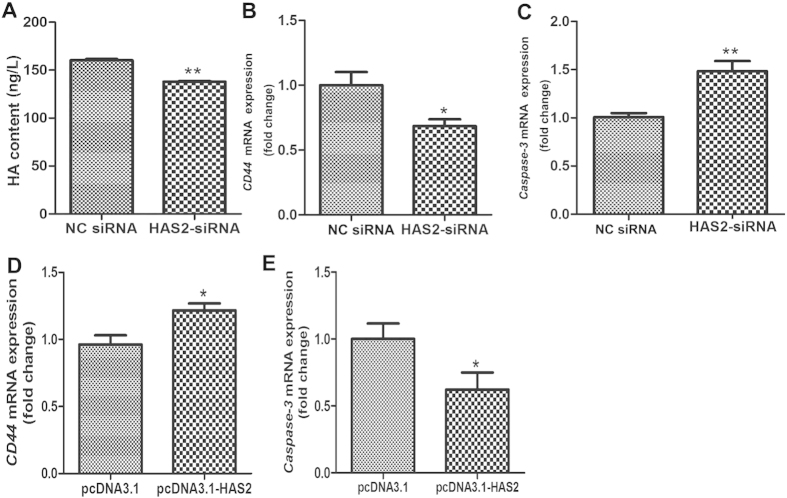
HAS2 regulates pGC apoptosis via the HA-CD44-Caspase-3 pathway. (**A**) Knockdown of HAS2 affected HA content in pGCs. mRNA expression of CD44 (**B**) and Caspase-3 (**C**) after siRNA-mediated knockdown of HAS2. (**D**) Overexpression of HAS2 significantly increased CD44 mRNA expression. (**E**) Overexpression of HAS2 significantly decreased Caspase-3 mRNA expression. *P < 0.05, **P < 0.01.

HAS2 is a key enzyme for HA content, while HA inhibits pGC apoptosis via CD44 and represses Caspase-3 expression, a marker of apoptosis^3^,^4^. To determine the mechanism by which HAS2 regulates pGC apoptosis, we examined the level of HA and expression of CD44 and Caspase-3 in pGCs treated with HAS2-siRNA. As expected, HA content was disturbed in pGCs treated with HAS2-targeting siRNA (Fig. 6A). Meanwhile, RT-qPCR analysis showed that the mRNA level of CD44 was significantly decreased by HAS2-siRNA treatment (Fig. 6B), whereas the mRNA level of Caspase-3 was significantly increased (Fig. 6C). In addition, overexpression of HAS2 induced the expression of CD44 (Fig. 6D), and reduced the expression of Caspase-3 (Fig. 6E). These results indicate that HAS2 regulates pGC apoptosis through the HA-CD44-Caspase-3 pathway and define a novel pathway, HAS2-HA-CD44-Caspase-3.

### miR-26b regulates the HAS2-HA-CD44-Caspase-3 pathway

**Figure 7:**
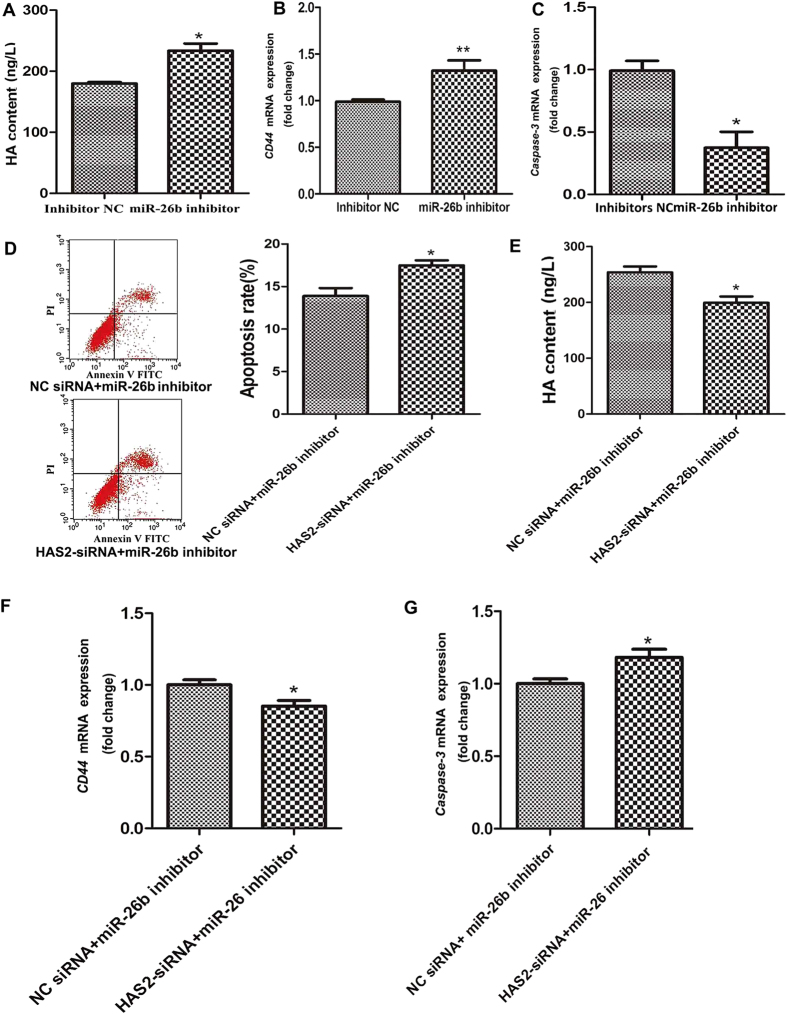
miR-26b regulates pGC apoptosis via the HAS2-HA-CD44-Caspase-3 pathway. (**A**) Transfection of a miR-26b inhibitor significantly increased HA content. HA content was detected by ELISA. mRNA expression of CD44 (**B**) and Caspase-3 (**C**) was increased and decreased, respectively, by the miR-26b inhibitor. (**D**) The apoptosis rate of pGCs co-transfected with a miR-26b inhibitor and HAS2-siRNA or NC siRNA. (**E**) HA content was detected by ELISA after co-transfection of a miR-26b inhibitor and HAS2-siRNA or NC siRNA. CD44 (**F**) and Caspase-3 (**G**) mRNAs were detected following the same treatment. The average results from three independent experiments are shown. *P < 0.05, **P < 0.01.

Our previous data revealed that HAS2, the initial signaling molecule in the HAS2-HA-CD44-Caspase-3 pathway, is a target of miR-26b in pGCs. To determine whether miR-26b regulates the HAS2-HA-CD44-Caspase-3 pathway, we monitored changes in this pathway in pGCs transfected with a miR-26b inhibitor. In contrast to the effects of HAS2-siRNA treatment, knockdown of miR-26b significantly enhanced HA content in pGCs (Fig. 7A). Meanwhile, knockdown of miR-26b increased the CD44 mRNA level (Fig. 7B) and significantly decreased the Caspase-3 mRNA level (Fig. 7C). This showed that miR-26b regulates the HAS2-HA-CD44-Caspase-3 pathway in pGCs by targeting HAS2.

### HAS2 reduces the role of miR-26b in pGCs

To further validate the importance of HAS2 in miR-26b regulation of pGC apoptosis and the HAS2-HA-CD44-Caspase-3 pathway, we knocked down both miR-26b and HAS2 in pGCs by co-transfecting a miR-26b inhibitor and HAS2-siRNA. The apoptosis rate was significantly higher in pGCs co-transfected with a miR-26b inhibitor and HAS2-siRNA than in pGCs transfected with a miR-26b inhibitor and NC siRNA (Fig. 7D). Moreover, in pGCs co-transfected with a miR-26b inhibitor and HAS2-siRNA, the HA content (Fig. 7E) and CD44 mRNA level (Fig. 7F) were decreased, while the Caspase-3 mRNA level was increased (Fig. 7G). Together, these results show that miR-26b regulates pGC apoptosis and the HAS2-HA-CD44-Caspase-3 pathway by targeting HAS2.

### HA prevents pGC apoptosis caused by HAS2-siRNA or miR-26b

**Figure 8:**
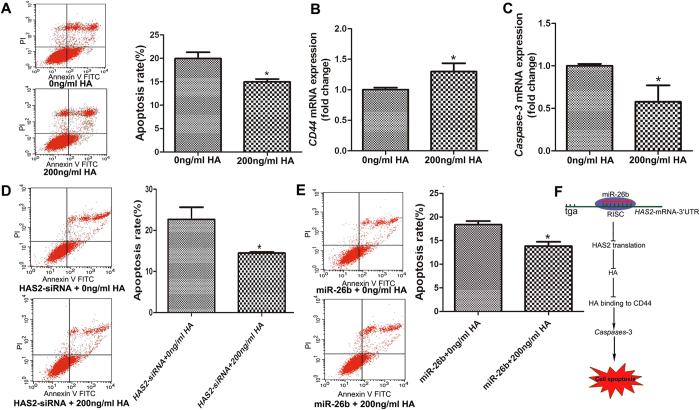
HA prevents pGC apoptosis caused by HAS2-siRNA or miR-26b. (**A**) HA inhibits pGC apoptosis. High molecular weight HA (HMW~2 × 10^6^ Da) was added to the culture media. (**B**) HA promotes the expression of CD44. (**C**) HA decreased the expression of Caspase-3. (**D**) HA rescued pGC apoptosis caused by HAS2-siRNA. (**E**) HA rescue pGC apoptosis caused by miR-26b. (**F**) Model of the miR-26b-mediated HAS2-HA-CD44-Caspase pathway in the regulation of pGC apoptosis. The average results from three independent experiments are shown. *P < 0.05.

To confirm the anti-apoptotic role of HA in pGCs, high molecular weight HA (HMW~2 × 10^6^ Da) was added to the culture media. The addition of HA inhibited pGC apoptosis compared with the untreated control, indicating that HA has an inhibitory effect on pGC apoptosis (Fig. 8A). In addition, HA promoted the expression of CD44 (Fig. 8B), and suppressed the expression of Caspase-3 (Fig. 8C) in pGCs. To further explore whether HA mediates the function of miR-26b and the HAS2 axis in pGC apoptosis, we co-treated pGCs with HA in the presence of HAS2-siRNA or miR-26b. As expected, HA addition rescued pGC apoptosis caused by HAS2-siRNA (Fig. 8D) or miR-26b (Fig. 8E).

## Discussion

Apoptosis is a complex process regulated by multiple factors and signal transduction pathways^30^. Recently, an increasing number of studies indicate that miRNAs play important roles in the regulation of apoptosis^31^,^32^. Here, we demonstrated that miR-26b is a pro-apoptotic factor in pGCs. Moreover, we showed that HAS2, which is downregulated during porcine ovarian follicular atresia, is a direct target of miR-26b in pGCs and inhibits pGC apoptosis. miR-26b promotes pGC apoptosis through the HAS2-HA-CD44-Caspase-3 pathway by repressing HAS2 translation.

miRNAs are small non-coding RNAs that degrade mRNAs or inhibit mRNA translation at the post-transcriptional level^31^. Many miRNAs are clustered together on the genome; these clusters are referred to as miRNA clusters or families, such as the miR-26 family^33^,^34^. The human and mouse miR-26 family has three members: miR-26a-1, miR-26a-2, and miR-26b. Each of these is embedded within the introns of genes encoding proteins belonging to the carboxyl-terminal domain RNA polymerase II polypeptide A small phosphatase (CTDSP) family, which includes CTDSPL, CTDSP2, and CTDSP1^34^. In cancer cells and normal liver tissues, miR-26a and miR-26b have a similar expression pattern, and both inhibit the G1/S transition by activating pRb protein^33^. In addition, our previous study showed that miR-26b is significantly upregulated during porcine follicular atresia, while miR-26a is not changed^28^. Herein, we identified and characterized pig miR-26b, and found that miR-26b is highly conserved among mammals. These results indicate that the precursor and mature sequences of miR-26b are highly conserved^35^ and that miR-26b has a similar function in different species.

miR-26b is one of the most studied miRNAs^36^,^37^ and plays an important role in the regulation of apoptosis^27^,^38^,^39^. miR-26b functions by targeting PFKFB3 to induce osteosarcoma cell apoptosis^39^, and promotes breast cancer cell apoptosis by targeting the SLC7A11 gene^40^. Consistently, the miR-26b level is positively correlated with the apoptosis rate in hepatocellular carcinoma tissues^41^. miR-26b plays an important role in mesangial cell apoptosis^42^, and increases apoptotic cell death in primary neuronal cultures by targeting Rb1^43^. All these data indicate that miR-26b is a pro-apoptotic miRNA. In the present study, inhibition of miR-26b reduced pGC apoptosis. Similarly, Lin *et al.*^28^ and Liu *et al.*^27^ reported that overexpression of miR-26b promotes pGC apoptosis. These data further demonstrate that miR-26b is involved in pGC apoptosis.

miRNAs usually function by targeting specific genes directly, and miR-26b is no exception. More than a dozen genes, which are linked with a variety of cell types and functions, have been confirmed as direct miR-26b target genes. For example, miR-26b regulates prostatic branching by targeting Hgf^44^, neural stem cell differentiation by targeting ctdsp2^45^, adipocyte differentiation and proliferation by targeting Smad1^46^, cardiomyocyte apoptosis by targeting PTEN^47^, cancer cell processes by targeting CTDSP2^33^ and PFKFB3^39^, and virus transcription and replication by targeting CHORDC1^48^. Similarly, miR-26b promotes ovarian GC apoptosis by targeting various genes such as ATM^28^, Smad4^27^, and USP9X^49^. Here, we identified HAS2, a key enzyme for HA synthesis, as a novel target of miR-26b in pGCs. The HAS2 mRNA level is lower in ovarian GCs of PCOS patients^14^ and higher in human GCs treated with hCG^15^. hCG treatment significantly decreases GC apoptosis^50^ and increases GC viability^29^. FSH, which functions as an apoptosis suppressor, increases the HAS2 mRNA level^51^. Downregulation of HAS2 can arrest pGCs in S phase of the cell cycle, which indicates that HAS2 plays an important role in GC function^13^. We demonstrated that knockdown of HAS2 enhanced pGC apoptosis, while overexpression of HAS2 decreased pGC apoptosis, suggesting that HAS2 is an inhibitory regulator of pGC apoptosis and follicular atresia, and target regulated by miR-26b. In addition, HAS2 is also controlled by other miRNAs such as miR-23a-3p^26^ and let-7^51^. miR-23a-3p which decreases HAS2-mediated HA content, is positively correlated with fibroblast age and senescence, and HAS2 is a target gene of miR-23a-3p^26^. HAS2 has binding sites for let-7 family and the treatment of cells with anti-let-7 results in an increase in cell survival^52^.

In mammalian ovaries, HA plays an important role in follicular atresia and GC functions such as survival and apoptosis, and drive from HAS2^3^. Inhibition and activation of HA synthesis increases and decreases pGC apoptosis, respectively^3^,^4^. Meanwhile, KGN cells, a human ovarian GC line, and primary rat GCs treated with HA have a higher number of generations and a higher proliferation index than control cells^53^. HA elicits its biological effects mainly though binding to the extracellular N-terminal hyaluronan-binding domain of its membrane receptor CD44, a large transmembrane glycoprotein^54^,^55^. Inhibition and activation of HA content decreases and increases CD44 expression, respectively^4^. HA can bind to CD44 in order to inhibit the activation of Caspases, markers of apoptosis in pGCs, suggesting that HA-CD44 binding is important for apoptosis inhibition in pGCs^4^,^51^. In this study, we further confirmed that HA inhibits pGC apoptosis, and demonstrated that both miR-26b and HAS2 affected the activity of the HA-CD44-Caspase-3 pathway in pGCs. Therefore, we speculate that miR-26b regulates HA content and the HA-CD44-Caspase-3 pathway by targeting HAS2, resulting in pGC apoptosis and follicular atresia.

In conclusion, our data confirm that miR-26b enhances pGC apoptosis. HAS2 was identified as a novel target of miR-26b, and its protein level was downregulated by miR-26b. Moreover, this reduction in HAS2 is one mechanism by which miR-26b enhances pGC apoptosis. We revealed a novel pathway that controls GC apoptosis and follicular atresia in mammalian ovaries, the miR-26b-HAS2-HA-CD44-Caspase-3 pathway (Fig. 8F).

## Methods

### Ethics statement

All experiments were performed in accordance with the guidelines of the regional Animal Ethics Committee and were approved by the Institutional Animal Care and Use Committee of Nanjing Agricultural University.

#### Bioinformatic analysis

The precursor sequence of the pig miR-26b gene was isolated by PCR amplification and clone sequencing. The primers and amplification conditions are listed in Supplementary Table S1. Other vertebrate miR-26b sequences were obtained from miRBase (http://www.mirbase.org) and GenBank (http://www.ncbi.nlm.nih. gov/). Multiple sequence alignments were performed using Clustal X 1.83 software. The stem-loop structures of porcine pre-miR-26b sequences were predicted using the Vienna RNAfold webserver (http://rna.tbi.univie.ac.at/cgi-bin/RNAfold.cgi). The targets of miR-26b and miRNAs that target HAS2 were predicted by five algorithms: TargetScan, miRanda, PITA, DINA-mT, and RNAhybrid. miRNAs predicted to regulate pig HAS2 were compared using the publically available Gene List Venn Diagram (http://www.pangloss.com/seidel/Protocols/venn.cgi).

#### Cell culture and transfection

Cells (pGC and HeLa 229 cells) were cultured and transfected according to the methods described by Liu *et al.*^27^. A chemically synthesized miR-26b inhibitor, inhibitor NC, miR-26b mimics, NC mimics, HAS2-siRNA, siRNA NC. were obtained from GenePharma (Shanghai, China) (Supplementary Table S1). Cells were routinely serum starved before adding HA^4^. HA (HMW~2 × 10^6^ Da) was purchased from Sangon Biotech (Shang Hai, China).

#### RT-qPCR

Total RNA was isolated from pGCs using TRIzol reagent (Invitrogen, Shanghai, China) and reverse-transcribed into cDNA using the M-MLV reverse transcriptase and oligo (dT)_18_ primers. Relative expression levels according to the treatment were measured using SYBR Premix Ex Taq (Takara, Dalian, China) and calculated using the 2^−ΔΔCt^ method, with normalization to GAPDH as the endogenous control. The primer sequences are listed in Supplementary Table S2.

#### Apoptosis assay

The pGC apoptosis rate was determined using the Annexin V FITC/Propidium Iodide Kit (Vazyme Biotech, China), according to the manufacturer’s instructions. Cells were sorted by fluorescence-activated cell sorting using a Beckman Coulter instrument (Becton Dickinson, Franklin, NJ, USA).

#### Dual-luciferase assay

Fragments of the 3′ UTR of HAS2 containing the predicted miR-26b-binding site or with mutations in the putative miR-26b seed sequence were cloned and inserted between the *Sac*I and *Xho*I restriction sites of the pmirGLO Dual-Luciferase miRNA Target Expression Vector using the primers listed in Supplementary Table S2. The binding site was mutated from ATCCGA to CTGGTC. Luciferase activity was measured 24 h after transfection using the Dual-Glo luciferase assay system (Promega, USA). Renilla luciferase activity served as the internal control.

#### Western blotting

pGCs were collected using RIPA buffer supplemented with the protease inhibitor phenylmethylsulfonyl fluoride. Total protein concentrations were determined using the BCA Protein Assay Kit (Beyotime, China). Western blotting was performed as described previously^27^. The antibodies used were anti-HAS2 (Santa Cruz Biotechnology, USA) and anti-GAPDH (Cell Signaling Technology, USA). The HAS2 molecular mass is 63 KD. The GAPDH molecular mass is 37 KD. Cropped gels were used for western . Densitometric analysis was performed to quantify signal intensities using Image J software.

#### ELISA for Hyaluronan

An enzyme-linked immunosorbent assay (ELISA) was used to determine the concentration of HA in medium as described previously^9^,^56^,^57^. Culture media was collected after treatment and 96-well plates were precoated with HABC (a hyaluronan-binding complex). Plates was incubated overnight at 4 °C, washed with phosphate-buffered saline (PBS) containing 0.5% Tween, and blocked with PBS containing 1% bovine serum albumin. Samples were diluted 1:40, added to the wells, and incubated for 1 h at 37 °C. Hyaluronan standards were also added. Plates were washed with PBS containing 0.5% Tween and incubated with 1 μg/ml HABC at 37 °C for 1 h. Horseradish peroxidase-conjugated streptavidin was added to the wells and incubated for 1 h at 37 °C. Plates were incubated with substrate-chromogen solution. Absorbance at 450 nm was measured Absorbance at 450 nm was measured after the addition of 50 μl of 2 M H_2_SO_4_.

#### RNA interference

siRNA was used to knockdown HAS2 in pGCs. HAS2-siRNA (sense 5′-CCG GGU UCU UCC CUU UCU UTT-3′ and antisense 5′-AAG AAA GGG AAG AAC CCG GTT-3′) and NC siRNA (scramble, code 4611) were purchased from GenePharma. Transfection was performed with Lipofectamine^®^ 2000 (Invitrogen).

### Overexpression of HAS2

The cDNA sequence of HAS2 was content by Generay (Shanghai, China) using the mRNA sequence of pig HAS2 (GenBank accession no. NM_214053.1), inserted into the pcDNA3.1(+) vector, and sequence verified. Transfection was performed with Lipofectamine^®^ 2000 (Invitrogen).

### Statistical analysis

At least three biological replicates were used for each analysis. All results are reported as means ± standard error of the mean (SEM). Statistical analysis was performed with an analysis of variance (ANOVA) for multiple comparisons using SPSS 18.0 software (SPSS Inc., USA).

## Additional Information

**How to cite this article**: Liu, J. *et al.* Conserved miR-26b enhances ovarian granulosa cell apoptosis through HAS2-HA-CD44-Caspase-3 pathway by targeting HAS2. *Sci. Rep.* **6**, 21197; doi: 10.1038/srep21197 (2016).

## Supplementary Material

Supplementary Information
