# Supplementary material for: Conserved miR-26b enhances ovarian granulosa cell apoptosis through HAS2-HA-CD44-Caspase-3 pathway by targeting HAS2
Source: Sci Rep. 2016 Feb 18;6:21197. doi: 10.1038/srep21197 (PMC4758074; doi:10.1038/srep21197)
Supplement: Supplementary Information [file srep21197-s1.doc]

# Conserved miR-26b enhances ovarian granulosa cell apoptosis through HAS2-HA-CD44-Caspase-3 pathway by targeting HAS2

Jiying Liu^*^, Fei Tu^*^, Wang Yao, Xinyu Li, Zhuang Xie, Honglin Liu, Qifa Li^*^ and Zengxiang Pan^*^

College of Animal Science and Technology, Nanjing Agricultural University, Nanjing 210095, People’s Republic of China

^*^ These authors contributed equally to this work as co-first authors

Corresponding authors.

Fax: +86 025 8439 5314

E-mail address: liqifa@njau.edu.cn, owwa@njau.edu.cn

**Supplementary Data**


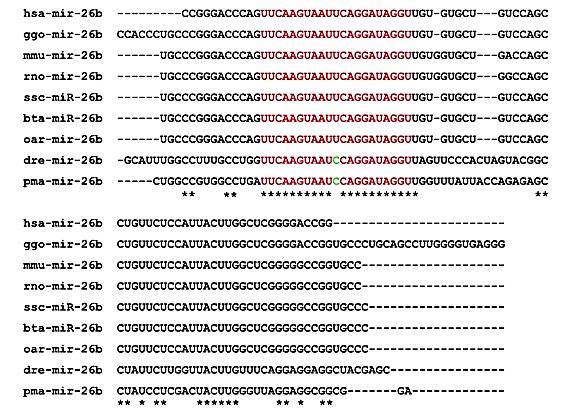


**Supplementary Figure S1. Characterization of porcine miR-26b precursor sequences.** The precursor of pig miR-26b was [amplified](http://dict.cn/amplified production) and aligned with those of miR-26b in other species. The miR-26b precursor is 85 bp long, which is highly consistent with other species. The [asterisk](http://dict.cn/asterisk) indicates complementarity. The red letters indicate the mature sequences of miR-26b in the precursor and the green letters show in Actinopterygii contains a U→C mutation.


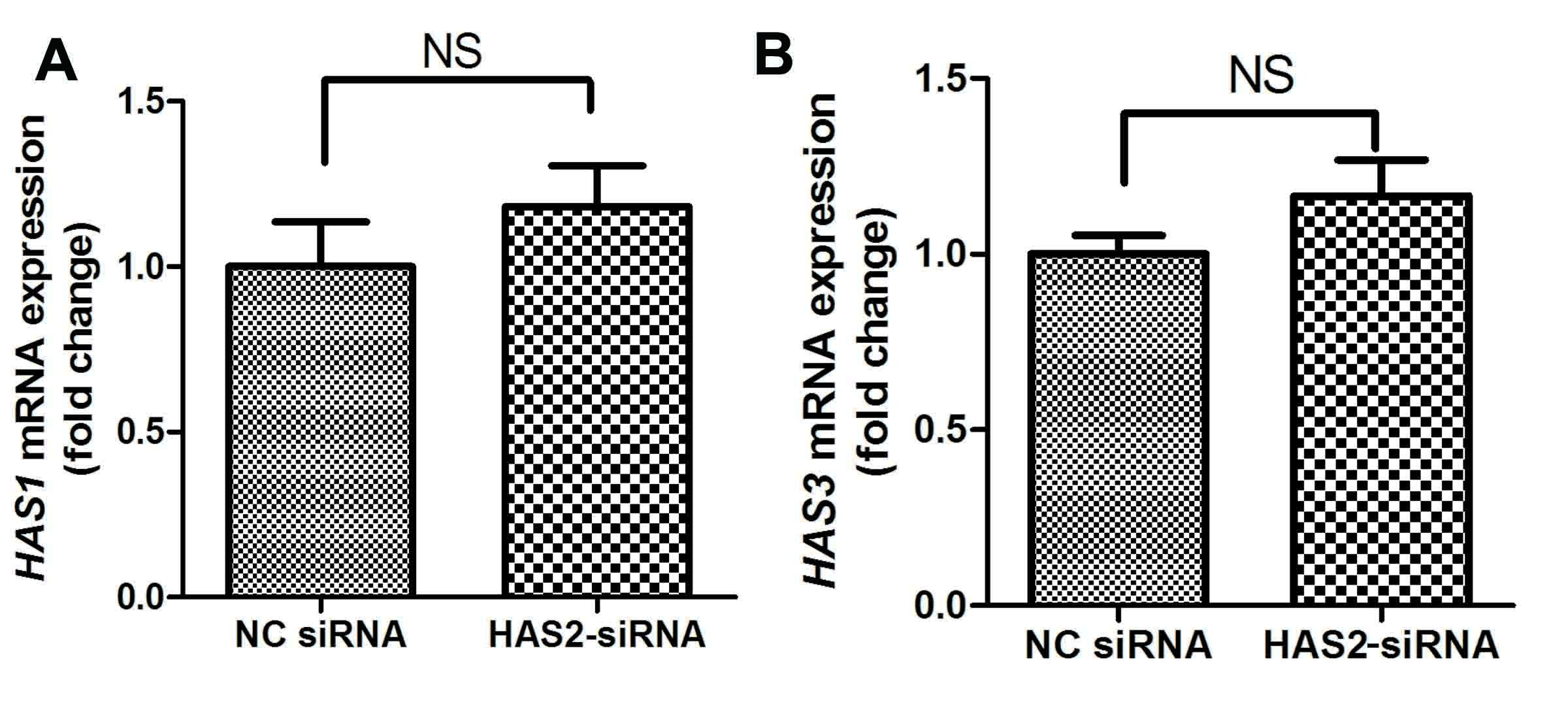


**Supplementary Figure S2. The mRNA expression of HAS1 and HAS2 after HAS2 was knockdown by HAS2- siRNA.** HAS2 was knockdown by HAS2- siRNA in pGC, the expression of HAS1 (A) and HAS3 (B) were upregulated but do not significant. NS: not significantly different at 5 % level.

**Supplementary Table S1**

**The sequences of miR-26b and miR-26b inhibitors used in this study.**

| Name | sequence（5’→3’） |
| --- | --- |
| mimics NC | F: UUC UCC GAA CGU GUC ACG UTT  R: ACG UGA CAC GUU CGG AGA ATT |
| miR-26b mimics | F:UUC AAG UAA UCC AGG AUA GGU  R:CUA UCC UGA AUU ACU UGA AUU |
| Inhibitors NC | CAG UAC UUU UGU GUA GUA CAA |
| miR-26b inhibitors | ACC UAU CCU GAA UUA CUU GAA |
| HAS2-siRNA | F: CCG GGU UCU UCC CUU UCU UTT  R: AAG AAA GGG AAG AAC CCG GTT |
| NC siRNA | F: UUC UCC GAA CGU GUC ACG UTT R: ACG UGA CAC GUU CGG AGA ATT |

**Supplementary Table S2**

**The primers used in this study.**

| Primer | Primer sequence（5’→3’） | Size | Tm(℃) | Usage |
| --- | --- | --- | --- | --- |
| HAS2 | F:TTATGGGCAGCCAATGTA  R:ACTTGCTCCAACGGGTCT | 159 | 58 | qRT-PCR |
| Caspase-3 | F:GACTGTGGGATTGAGACG  R:ACCCGAGTAAGAATGTGC | 212 | 58 | qRT-PCR |
| miR-26b  precursor | F: CAGTGAACAATGCCGACTTC  R: CCCTTCCTCCACCGTGAC | 499 | 56.5 | precursor  amplified |
| Bcl-2 | F:GAAACCCCTAGTGCCATCAA  R:GGGACGTCAGGTCACTGAAT | 196 | 60 | qRT-PCR |
| CD44 | F: GAGGCGGCCCTGAACATA  R: AAGGTATTAGGCAGGTCTGTGAC | 218 | 60 | qRT-PCR |
| HAS1 | F：CGTGCTGCGGCTCTTCTA  R：TGGTTCATGGTGGCGAGT | 201 | 59.5 | qRT-PCR |
| HAS3 | F：CAAGTCTTACTTCCGGGAGTG  R：CTGTCAGCAGGAAGAGGAGAAT | 173 | 59 | qRT-PCR |
| GAPDH | F:GGACTCATGACCACGGTCCAT  R:TCAGATCCACAACCGACACGT | 220 | 58 | qRT-PCR |
| HAS2 | F:CGAGCTCAAGGGACAACAGTACGAC(Sac1)  R:CCGCTCGAGTGATAACCCACATAAAGC(Xho1) | 375 | 56.4 | Vector  construction |
